# Les grosses bourses aiguës au centre hospitalier de Louga, Sénégal: aspects épidémiologiques, étiologiques et thérapeutiques

**DOI:** 10.11604/pamj.2016.24.214.9876

**Published:** 2016-07-12

**Authors:** Ibrahima Diabaté, Bouréima Ouédraogo, Mbaye Thiam

**Affiliations:** 1 Service d’Urologie, CHR Amadou Sakhir Mbaye de Louga, Sénégal; 2 Service de Chirurgie, CHR Amadou Sakhir MBaye de Louga, Sénégal

**Keywords:** Grosse bourse aiguë, orchi-épididymite aiguë, torsion du cordon spermatique, traumatisme des bourses, hernie inguinale étranglée, gangrène de fournier, traitement, Sénégal, Acute scrotal swelling, acute orchiepididymitis, torsion of the spermatic cord, scrotal trauma, strangulated inguinal hernia, Fournier's gangrene, treatment, Senegal

## Abstract

**Introduction:**

Les grosses bourses aiguës (GBA) sont des motifs fréquents de consultation en urgence. Cette étude vise à déterminer la fréquence hospitalière des GBA au Centre Hospitalier Régional Amadou Sakhir Mbaye (CHRASM) de Louga au Sénégal, à identifier les formes cliniques et à évaluer les modalités de prise en charge.

**Méthodes:**

Il s’agit d’une étude rétrospective, descriptive portant sur 114 patients reçus pour GBA au CHRASM de Louga, de mai 2010 à août 2013. Les variables étudiées étaient: la fréquence des GBA parmi les grosses bourses (GB) et les urgences urologiques, l’âge, le délai de consultation, les causes, le traitement, l’évolution post-thérapeutique et la durée d’hospitalisation.

**Résultats:**

Au cours de la période d’étude, 356 GB et 420 urgences urologiques ont été enregistrées. Ainsi, les 114 cas de GBA recensés représentaient 32,0 % des GB et 27,1 % des urgences urologiques. La moyenne d’âge était de 42,25 ± 25ans (extrêmes de 5 mois et 89 ans). Le délai moyen de consultation était de 4 jours. Le diagnostic évoqué à l’admission était: une orchi-épididymite aiguë (n=66), une GBA abcédée (n=18), une suspicion de torsion du cordon spermatique (n=14), une GBA traumatique (10 cas), une hernie inguino-scrotale étranglée (06 cas). Le traitement a été exclusivement médical dans 66 cas (57,8 %). L’exploration chirurgicale qui était indiquée chez 48 patients, a été effectuée chez 45 d’entre eux (93,7 %), trois patients (6,2 %) l’ont refusé. Au total, il y a eu 9 cas d’orchidectomies et 36 procédures conservatrices. La durée moyenne d’hospitalisation était de 3± 2 jours. Quatre vingt et un patients (71,0 %) ont été hospitalisés pendant 24 heures au moins; ils étaient répartis en 42 patients chirurgicaux et 39 patients médicaux. Aucun cas de décès n’a été enregistré.

**Conclusion:**

Les GBA sont fréquentes dans notre structure, tous les âges sont concernés. Elles sont dominées par les GBA d’origine infectieuse, les torsions du cordon spermatique et les traumatismes des bourses. Le retard à la consultation est souvent la règle, ce qui met en jeu le pronostic fonctionnel du testicule.

## Abstract (en)

**Introduction:**

Acute scrotal swellings (ASS) are a common reason for emergency consultation. This study aims to determine the frequency of hospitalization for ASS at the Louga Amadou Sakhir Mbaye Regional Hospital, Senegal, to identify its clinical forms and to evaluate the care management.

**Methods:**

This is a retrospective descriptive study involving 114 patients hospitalized for ASS at the Louga Amadou Sakhir Mbaye regional hospital, from May 2010 to August 2013. The variables studied were: the frequency of ASS among scrotal swellings and urological emergencies, age, consultation period, causes, treatment, post-treatment evolution and hospitalization length.

**Results:**

During the study period, 356 scrotal swellings and 420 urological emergencies were recorded. Thus, 114 cases with ASS accounted for 32.0% of scrotal swellings and 27.1% of urological emergencies. The average age was 42.25 ± 25 years (5 months and 89 years represent the extremes). The median of consultation time was 4 days. The diagnosis at admission was: acute orchiepididymitis (n=66), ASS with abscess formation (n=18), suspicion of torsion of the spermatic cord (n=14), traumatic ASS (10 cases), strangulated inguinoscrotal hernia (06 cases). Treatment was exclusively medical in 66 cases (57.8 %). Surgical exploration, which was indicated in 48 patients, was performed in 45 of them (93.7%), three patients (6.2%) refused it. In total there were 9 cases with orchiectomies and 36 conservative procedures. The average length of stay in hospital was 3 ± 2 days. Eighty-one patients (71.0%) were hospitalized for at least 24 hours. They were divided into two groups: 42 surgical patients and 39 medical patients. No deaths were recorded.

**Conclusion:**

ASS are common in our hospital, people of all ages can be affected. They are dominated by ASS of infectious origin, torsion of the spermatic cord and scrotal trauma. Delayed consultation is often the rule, this may affect functional prognosis of the testicle.

## Introduction

Les grosses bourses aiguës (GBA) sont des urgences non moins rares en urologie [1–3]. Elles regroupent toutes les bourses douloureuses, traumatiques ou non, avec ou sans lésion ouverte, infectées ou non. Elles ont en commun la douleur et la tuméfaction des bourses, posant ainsi un problème de diagnostic différentiel. Les pathologies le plus souvent en cause sont : la torsion du cordon spermatique (TCS), les traumatismes des bourses, les infections urogénitales (orchi-épididymite, abcès des bourses et gangrène de Fournier), la hernie inguino-scrotale étranglée (HISE), le tableau aigu du cancer du testicule [1–7]. Au regard de ces étiologies nombreuses, l’écho-doppler a pris de nos jours une place importante dans l’exploration des GBA [4, 7–9] pour lesquelles un diagnostic rapide s’impose afin de distinguer les cas justiciables d’une intervention chirurgicale. La TCS demeure parmi toutes ces GBA la seule dont la suspicion indique une exploration chirurgicale immédiate qui ne saurait être retardée par un quelconque bilan para-clinique, au risque d’une nécrose testiculaire au-delà de six heures. Le but de cette étude était de déterminer la fréquence hospitalière des GBA au Centre hospitalier régional Amadou Sakhir MBaye (CHRASM) de Louga au Sénégal, d’identifier les différentes formes cliniques et d’évaluer les modalités de prise en charge.

## Méthodes

Il s’agit d’une étude rétrospective et descriptive réalisée au CHRASM de Louga (Sénégal) de mai 2010 à août 2013 soit 39 mois. Elle a porté sur 114 patients reçus et pris en charge pour une GBA. Tous les cas ont été reçus soit au service des urgences dudit centre puis vus en consultation d’urologie, soit directement reçus en consultation urologique. La collecte des données a été faite sur des fiches préétablies à partir des dossiers des patients, des registres de consultations urologiques et d’urgences urologiques, des registres d’hospitalisation et de compte rendu opératoire. Le traitement des données a été fait à l’aide des logiciels Microsoft Office Word et Excel 2007 pour en tirer des moyennes, des écart-types et des extrêmes. Les variables qui ont été étudiées étaient : la fréquence des GBA par rapport aux grosses bourses (GB) et aux urgences urologiques, l’âge des patients, le délai avant la consultation, les causes des GBA, les éléments du diagnostic (clinique, para-clinique et opératoire), le mode de prise en charge, le traitement et l’évolution post-thérapeutique. Dans le cadre du bilan para-clinique à la recherche d’une co-morbidité, il a été réalisé chez certains patients une glycémie ou une sérologie rétrovirale HIV (Human Immunodefiscience Virus) après l’obtention d’un consentement libre et éclairé.

## Résultats

Durant cette période d’étude, il a été répertorié 356 grosses bourses (GB) et 420 urgences urologiques. Ainsi, les 114 cas de GBA représentaient 32,0 % des GB et 27,1 % des urgences urologiques. La moyenne d’âge dans l’ensemble était de 42,25 ± 25 ans avec des extrêmes de 5 mois et 89 ans. La tranche d’âge la plus touchée était celle de 61 ans et plus (39 %), suivie de celle de 16 à 30 ans (26,5 %). Le délai moyen avant la consultation était de 3 jours (extrêmes 2 heures et 13 jours) mais inégal selon la cause de la GBA (Tableau 1). Les diagnostics retenus à l’admission étaient de 66 cas (57,9 %) d’orchi-épididymites aiguës (OEA), les autres 48 cas (42,1 %) étaient des GBA abcédées, des TCS, des GBA traumatiques et des HISE (Tableau 1). L’échographie a été pratiquée uniquement chez 55 patients répartis en : 31 cas de OEA, 10 cas de GBA abcédées, 4 cas de GBA traumatiques et 10 cas de TCS présumée. L’OEA se caractérisait par un épididyme hyperéchogène et augmenté de volume, une lame d’hydrocèle et un épaississement des enveloppes. Dans les cas de GBA abcédées, on notait un épaississement des tuniques des bourses avec des plages hypo ou anéchogènes. Concernant les GBA traumatiques, il a été noté une rupture de la continuité de l’albuginée dans un cas et un hématome des bourses pour les autres cas. Dans les TCS, les images étaient variables, hypo ou hyperéchogènes avec un épaississement du scrotum. Par ailleurs, la glycémie et la sérologie rétrovirale HIV ont été pratiquées en urgence respectivement dans 26 cas et 9 cas qui présentaient un tableau infectieux sévère. Trois cas de diabète et un cas de HIV positif ont été décelés. Sur le plan étiopathogénique, le Tableau 2 présente les différentes causes des GBA infectieuses, essentiellement dominées par les tumeurs prostatiques et les infections urétro-prostatiques.

**Tableau 1:** Les GBA selon la moyenne d’âge et les délais moyens de consultation

Grosses bourses aigues	Nombre de cas (%)	Moyenne d’âge	Délai moyen de consultation
Orchiépididymite aiguë	66 (57,89%)	52,26	72,00 ± 18,72 Heures
Bourse aiguë abcédée	18 (15,78%)	57,55	10,29 ± 01,72 Jours
Torsion cordon spermatique	14 (12,28%)	26,57	33,14 ± 20,50 Heures
Bourse aiguë traumatique	10 (08,77%)	15,77	28,90 ± 23,22 Heures
Hernie inguinoscrotale étranglée	6 (05,26%)	11,16	07,50 ± 05,31 Heures
Total	114 (100%)		

**Tableau 2:** Etiologies des GBA infectieuses

Affections	Orchiépididymites aiguës	Bourses aiguës abcédées
Infection urétro-prostatique	15	07
Sonde à demeure	10	03
Tumeur prostatique	27	00
Sténose de l’urètre	00	04
Origine anale	00	02
Cause indéterminée	14	02
**Total**	**66**	**18**

Le traitement a été exclusivement médical pour 66 patients (57,9 %) repartis en OEA (n=64) et GBA traumatique (n=2). L’indication opératoire a été posée chez 48 patients (42,1 %) sur 114. Quarante cinq patients (93,7 %) l’ont accepté contre 3 cas de refus (6,2%) repartis 2 cas de suspicion de TCS et un cas de GBA traumatique. Le diagnostic préopératoire a été infirmé dans 2 cas explorés chirurgicalement pour suspicion de TCS. Il s’agissait en réalité d’un cas de torsion d’hydatide de Morgani et d’un cas d’œdème aigu idiopathique des bourses. Par ailleurs, dans l’un des cas de HISE, il a été observé une nécrose ischémique du testicule du côté de la hernie étranglée. Le traitement chirurgical, en fonction des pathologies évoquées à l’admission et des lésions observées en per-opératoire se résumait comme suit: dans les GBA abcédées, il a été réalisé une orchidectomie pour fonte purulente du testicule (n=2) ; un drainage chirurgical d’abcès (n=14); un débridement pour gangrène de Fournier (n=4). Une cystostomie complémentaire (au drainage d’abcès et au débridement) a été pratiquée dans 7 cas, en raison d’une sténose urétrale associée; dans les TCS, ont été réalisées une orchidectomie pour nécrose ischémique du testicule associée à une orchidopexie du testicule controlatéral (n=5) ; une orchidopexie bilatérale pour TCS unilatérale avec un testicule viable après détorsion (n=5) ; une résection d’hydatide de Morgani (n=1) ; enfin aucun geste n’a été fait lors de la scrototomie pour œdème aigu idiopathique des bourses (n=1); concernant les GBA traumatiques, il a été pratiqué une orchidectomie pour une rupture et fracture testiculaire (n=1) ; un drainage chirurgical avec hémostase pour hématome des bourses (n=2) ; un parage et suture de plaie scrotale (n=4); dans les cas de HISE, les anses intestinales herniées étaient viables et la cure de la hernie a été faite avec fermeture du canal péritonéo-vaginal (n=6); une orchidectomie (n=1) a été associée dans l’un des 6 cas, pour nécrose ischémique du testicule du côté de la hernie étranglée. L’évolution au cours du traitement a été favorable dans la plupart des cas ; seulement 2 cas d’OEA (malgré le traitement médical) ont évolué en abcès et ont donc été drainés chirurgicalement. Les sujets diabétiques ont été équilibrés par insulinothérapie et le patient avec HIV positif a été mis sous trithérapie antirétrovirale.

Quatre vingt et un patients (71,0 %) ont été hospitalisés pendant 24 heures au moins. Ils étaient répartis en 42 cas chirurgicaux et 39 cas médicaux. La durée moyenne de séjour était de 3 ± 2 jours. Aucun cas de décès n’a été enregistré. Seuls 63 patients ont été revus sur une période de 6 mois dans le cadre du suivi et de la prise en charge chirurgicale de la maladie causale (tumeurs prostatiques, sténoses de l’urètre). Aucune plainte particulière au niveau des bourses ne nous a été rapportée.

## Discussion

Cette étude montre en suffisance, sur le plan de la fréquence la place qu’occupent les GBA dans notre institution: soit 32,0 % des GB et 27,1 % des urgences urologiques. Pour Moby Mpa et al [2] au Cameroun, la proportion des GBA atteind 71,13 % des urgences andrologiques alors que pour Fall et al [1] au Sénégal, elles se situent à seulement 9,13 % des urgences urologiques.

Les GBA regroupent diverses pathologies [1–7]. Toutefois dans cette série tout comme dans d’autres, il n’a été enregistré aucun cas de cancer du testicule dans sa forme aiguë (Tableau 3). Bien que tous les âges soient concernés, la moyenne d’âge dans cette série était de 42,25 ± 25 et objectivement liée à la forte représentation des personnes du troisième âge. Cette moyenne d’âge était différente d’une pathologie à une autre (Tableau 1) et il est aisé de constater que les adolescents et les adultes jeunes sont surtout concernés par les TCS, les GBA traumatiques et les HISE. Ces mêmes constatations avaient déjà été faites dans des études antérieures [1, 9–11]. Le délai avant la consultation, variable selon la pathologie en cause (Tableau 1) était relativement court pour les GBA à tableau clinique bruyant et de survenue brutale (TCS, GBA traumatique, HISE). Odzébe et al [9] affirmaient avoir reçu des cas de GBA traumatiques après une dizaine de jours. En revanche, les pathologies infectieuses (OEA, GBA abcédée et gangrène de Fournier) sont couramment reçues après plusieurs jours d’évolution. Dans notre étude, des cas ont même été reçus après 13 jours. Ce retard à la consultation serait lié aux mauvaises conditions socio-économiques [4, 6–8, 12, 13]: la pauvreté, l’automédication, le recours aux guérisseurs, la méconnaissance des pathologies des bourses. A tout cela, il faut aussi ajouter la négligence.

**Tableau 3:** Les GBA selon différentes séries

Grosses bourses aiguës	Moby et all [2] (n=207)	Fall et al [1] (n=113)	Notre série (n=114)
Orchiépididymite aiguë	30	-	66
Grosse bourse aiguë abcédée	39	51	18
Torsion du cordon spermatique	33	35	14^+^
Grosse bourse aiguë traumatique	105	27	10
Hernie inguino-scrotale étranglée	-	-	06

14^+^ : il s’agissait de 14 cas de suspicion de TCS, mais seulement 10 cas ont été confirmés à l’exploration chirurgicale

Le diagnostic des GBA repose sur la clinique. Toutefois, depuis plusieurs années, l’écho-doppler s’est imposé comme l’examen de choix des GBA [4, 7–9]. Elle permettrait de renseigner sur l’intégrité du bloc épididymo-testiculaire, le flux sanguin, la présence d’hématome, d’autres collections ou de corps étrangers [3, 4, 7, 9], de diminuer considérablement le nombre de scrototomie exploratrice ainsi que la durée d’hospitalisation [7, 14]. Aucun écho-doppler n’a été pratiqué dans notre série, faute d’équipement alors que dans celle de Khaleghnejad-Tabari et al [4], portant sur 100 cas de GBA, il n’a été réalisé que dans 9% des cas. Quant à l’échographie, elle a été pratiquée chez 48, 2 % (n=55) de nos patients, alors que chez Gnassigbé et al [6], ce taux n’était que de 21,5 %. D’autres examens, en particulier la glycémie et la sérologie HIV ont été initiées dans cette série, tout comme dans d’autres, à la recherche de facteurs de co-morbidité particulièrement dans les cas infectieux [12, 13, 15].

### La TCS

Elle est la seule réelle urgence chirurgicale parmi les GBA [6, 7, 11, 14–16]. C’est ainsi que le dogme de l’exploration chirurgicale systématique de toute GBA suspecte de TCS est toujours d’actualité dans notre institution même si ailleurs, les progrès de l’imagerie incitent à la modération [3, 5–7, 10, 14]. Des erreurs diagnostiques sont possibles. Elles sont découvertes à l’exploration chirurgicale et sont sans conséquence. Nous en avons eu dans notre série où, sur 12 patients explorés chirurgicalement pour suspicion de TCS, dix cas (n=10) ont été confirmés contre un cas de torsion d’hydatide de Morgani et un cas d’œdème aigu idiopathique des bourses [7]. Kaboré et al [8] au Burkina Faso, dans une série de scrototomie pour suspicion de TCS, ont rapporté 21,6 % d’OEA. L’orchidectomie indiquée dans la nécrose ischémique du testicule, a été réalisée dans 50 % des cas de TCS dans notre série. Ce taux atteint 55% dans la série de Kaboré et al [8] alors que Tajchner et al [3] n’ont rapporté qu’un cas d’orchidectomie pour 41 cas de TCS colligés en 10 ans. Quant à Gnassigbé et al [6], ils n’ont réalisé aucune orchidectomie dans une série de 17 cas de TCS.

### Les GBA traumatiques

Elles sont rarement rencontrées [3, 5, 9]. En 39 mois, nous n’avons enregistré que 10 cas (soit 8,77 % des GBA de notre série). Eriki et al [5], en cinq ans, n’ont enregistré aucun cas de traumatisme des bourses sur une série de 50 cas de GBA ; tandis que Odzébé et al [9] ont rapporté 18 cas en 17 ans. Cette rareté s’explique par des raisons anatomiques : la mobilité des testicules, le reflexe crémastérien, l’épaisseur et la résistance de l’albuginée testiculaire. Au plan lésionnel, les plaies et avulsions scrotales peuvent être observées tout comme des hématomes du scrotum, des hématocèles et des ruptures testiculaires. Si les parages et sutures suffisent pour ces plaies et avulsions scrotales, pour les hématomes et les ruptures testiculaires, l’exploration chirurgicale permet de vider la collection sanguine et de faire l’hémostase voire l’orchidectomie. Le traitement médical garde tout de même sa place dans les cas d’hématome des bourses sans lésion de l’albuginée testiculaire. C’est ainsi que sur une série de 18 patients, Odzébé et al [9] ont traité médicalement 6 patients (33,3 %) et chirurgicalement les 12 autres (66,6 %). Ils ont réalisé trois orchidectomies (n=3) et ont été conservateurs dans 9 cas. Dans notre série, 7 cas de GBA traumatiques ont été traités chirurgicalement avec un seul cas d’orchidectomie.

### Les HISE

La hantise dans les HISE demeure le risque de nécrose ischémique intestinale ou testiculaire dans une moindre mesure. Dans la première hypothèse, la résection intestinale avec anastomose termino-terminale s’impose alors que dans la seconde, c’est l’orchidectomie. Dans la présente étude, aucune nécrose intestinale n’a été constatée, cependant un cas de nécrose testiculaire a été enregistré et a nécessité une orchidectomie.

### Les GBA abcédées

Les GBA abcédées sont essentiellement les abcès compliquant les orchiépididymites, les fasciites nécrosantes des bourses ou gangrène de Founier. Le tableau clinique est habituellement sévère et exige une prise en charge pluridisciplinaire. En complément au drainage et au débridement, nous avons été amenés à pratiquer une cystostomie dans 7 cas pour une sténose urétrale associée. Benjeloun et al [12], dans une série de 50 cas de gangrène des organes génitaux externes ont pratiqué une colostomie dans 10 % des cas en raison d’une étiologie ano-rectale.

### Les OEA

Contrairement à quelques auteurs [1, 2] les OEA occupent la première place dans un certain nombre de travaux [4, 5] avec une incidence qui varie entre 28 % et 70 % selon les explorations [7]. Elles sont particulièrement fréquentes chez l’adulte où prédominent les infections bactériennes épididymo-testiculaires rétrogrades, d’origine vésicale, urétrale ou prostatique. Le traitement est basé sur une antibiothérapie de trois semaines [7], la suspension des bourses, les anti-inflammatoires non stéroïdiens et les antalgiques. L’évolution peut se faire vers la guérison, la formation d’abcès ou l’apparition de séquelles. Dans notre série, 2 cas sur un total de 66 cas ont évolué vers l’abcédation malgré le traitement médical institué.

## Conclusion

Les GBA sont des pathologies fréquentes dans notre structure hospitalière et tous les âges sont concernés. Les étiologies sont nombreuses, dominées essentiellement par les infections urogénitales, les TCS et les traumatismes. Le retard à la consultation est souvent la règle, ce qui met en jeu le pronostic fonctionnel du testicule quelque soit le traitement mis en route.

### Etat des connaissances actuelles sur le sujet

- Les GBA sont des urgences urologiques pouvant engager le pronostic fonctionnel des testicules;
- Elles présentent une grande diversité étiologique avec des prises en charge différentes.

### Contribution de notre étude à la connaissance

- Cet article révèle la place des GBA par rapport aux urgences urologiques en général et aux GB en particulier, dans un hôpital où elles sont étudiées pour la première fois.
